# Intra-tumoural microenvironment and bugs-based drug design: foreseeable future in oncology and immuno-oncology

**DOI:** 10.3389/fphar.2026.1732712

**Published:** 2026-01-30

**Authors:** Karolina Kaźmierczak-Siedlecka, Robert Kucharski, Ewa Stachowska, Iwona Pelikant-Małecka, Luigi Marano, Wojciech Makarewicz, Magdalena Kalinowska, Žilvinas Dambrauskas, Leszek Kalinowski

**Affiliations:** 1 Department of Medical Laboratory Diagnostics – Fahrenheit Biobank BBMRI.pl, Medical University of Gdansk, Gdansk, Poland; 2 Neodentica Dentistry Center, Gdansk, Poland; 3 Department of Human Nutrition and Metabolomics, Pomeranian Medical University in Szczecin, Szczecin, Poland; 4 Academy of Medical and Social Applied Sciences, Elbląg, Poland; 5 2nd Division of Radiology, Medical University of Gdansk, Gdańsk, Poland; 6 University of Social Sciences and Humanities, Warsaw, Poland; 7 Scientific Circle of Studies Regarding Personalized Medicine Associated with Department of Medical Laboratory Diagnostics, Medical University of Gdansk, Gdansk, Poland; 8 Department of Surgery, Lithuanian University of Health Sciences, Kaunas, Lithuania; 9 5BioTechMed Center, Department of Mechanics of Materials and Structures, Gdansk University of Technology, Gdansk, Poland

**Keywords:** drug design, engineered exosomes, immune system, intratumoural microbiome, microbiome, organoid-immune co-culture models, pharmacomicrobiomics, precision medicine

## Abstract

The term tumour microenvironment (TME) encompasses the coexistence of microorganisms and different cellular elements including endothelial cells, macrophages, cancer-associated fibroblasts and a complex network of microvessels. Integration of tumour immunity and intratumoural microbiome into anti-cancer strategies represents a promising frontier in precision oncology (for instance in case of solid cancers, such as pancreatic or colorectal tumours). Characterization of the intratumoural microbial signature has emerged as a critical step in drug discovery, influencing therapeutic efficacy as well as resistance. There are several approaches, such as elimination of pathogenic microorganisms within the TME, modulation of specific microbial–immune axes, including interactions among microbial species that may enhance or suppress tumour progression, and exploitation of bacterial strains engineered to express pro-drug-converting enzymes for localized tumour therapy *via* intratumoural injection. Furthermore, tumour organoid–immune co-culture models, particularly when combined with 3D bioprinting technologies, offer robust experimental platforms for dissecting tumour–microbiome–immune crosstalk. The reciprocal communication between the immune system and the tumour-associated microbiome/metabolome highlights novel opportunities for therapeutic innovation in oncology and immuno-oncology.

## Highlights

- The tumour microenvironment (TME) and its resident microbes shape the immune landscape of cancers and provide a foundation for novel anti-cancer drug design.
- Both immune-checkpoint inhibitors (ICIs) and non-checkpoint-based immunotherapies (e.g. personalized dendritic-cell vaccines) can be influenced, either enhanced or suppressed, by specific bacterial taxa.
- Innovative microbiome-based interventions include antigen-engineered commensal bacteria, bacteriophages targeting intratumoural pathogens, and direct intratumoural administration of bacterial strains with therapeutic potential.
- Spatially resolved transcriptomics (SRT), combined with 3D bioprinting, provides a powerful analytical framework for next-generation anti-cancer drug development.

## 1 Introduction

The complex interplay among the microbiome, metabolome, tumour microenvironment (TME) and immune system has recently become a central topic in cancer biology. This multidimensional crosstalk influences tumour initiation, progression, and therapeutic responses (Gopalakrishnan et al., 2018; Xu et al., 2024; Jiang H. et al., 2024; El Tekle and Garrett, 2023; Zhou et al., 2025; Galeano Niño et al., 2022; Fernandez et al., 2025; Park et al., 2022; Hu et al., 2025). Both bacterial and fungal components of the tumour microbiome modulate the immunophenotype of various cancers - a relationship confirmed through beta-diversity analyses, which reveal higher microbial richness in high-immunity tumours compared with low-immunity counterparts (Sheng et al., 2024). Local mucosal immunity, particularly within the intestine, is shaped by pattern recognition receptors (PRRs) on epithelial cells that detect pathogen-associated molecular patterns (PAMPs). These pathways are further influenced by key microbiota-derived metabolites, such as short-chain fatty acids (SCFAs), which enhance mucosal immunoglobulin A (IgA) production, regulate regulatory T cell (Treg) differentiation from naïve CD4^+^ T cells, and thus sustain immunological tolerance (Li Q. et al., 2024).

A balanced SCFAs ratio (acetate: propionate: butyrate ≈ 3 : 1: 1) is critical for intestinal homeostasis. Notably, the ratio between above mentioned SCFAs strongly depends on diet, gut region, the presence of disorders, as well as methodological aspects regarding measurement methods (Du et al., 2024; Li et al., 2025; Kaźmierczak-Siedlecka et al., 2022a). SCFAs are involved in the link between nutrition, microbiome, immunity, metabolism, and TME (Li et al., 2025). We previously reported (Kaźmierczak-Siedlecka et al., 2023a) that this proportion was disrupted in all patients with colorectal cancer (CRC) pre-operatively, with butyrate levels below normal in > 90% of cases. Butyrate, a principal product of bacterial fermentation, exerts multiple immunomodulatory effects, enhancing CD8^+^ T-cell-mediated anti-tumour activity, preserving T helper (Th) 17 cells/Treg balance, upregulating anti-inflammatory cytokines (e.g. interleukin 10 [IL-10]), downregulating pro-inflammatory mediators (e.g. interleukin 2 [IL-2], interferon gamma [IFN-γ]), and dendritic-cells maturation (Kaźmierczak-Siedlecka et al., 2022a; Abenavoli et al., 2023).

Given the microbiome’s ability to shape immune responses, its integration into oncological and immuno-oncological paradigms is both rational and necessary. There is also the term pharmacomicrobiomics, which is a field describing the interaction between microbiome and drugs regarding individual aspects. This bidirectional communication drug–microbiota is observed in different levels regarding drug bioavailability, effectiveness, and toxicity. Microbiota influences pharmacokinetics and pharmacodynamics of drugs (Gronich et al., 2025). The response to anti-cancer drugs can be modulated by gut microbiome either directly (by enzymatic activities) or indirectly (host-mediated both immune and metabolic mechanisms), whereas medications affect microbiome directly (through altering microbial growth and causing functional changes) (Bolte et al., 2025). Thus, pharmacomicrobiomics is strongly involved in precision cancer therapy allowing to optimize anti-cancer approaches (Ngoc et al., 2024; Kaźmierczak-Siedlecka et al., 2023b). Understanding the bidirectional communication between the immune system and the oral, gut, and tumour-associated microbiome is fundamental to develop multidisciplinary, mechanism-driven cancer therapies. In this paper, we discuss key molecular pathways at the interface of the TME and microbiome, explore their translational and clinical implications, and propose future perspectives in areas, such as microbiome-driven drug design, tumour organoid–immune co-culture systems, engineered exosomes, precision medicine, and spatially resolved transcriptomics (SRT).

## 2 Tumourigenesis–Tumour immunity and intratumoural microbes

The functioning of the immune system relies on a complex network of mediators. Among them, chemokines (small secreted proteins of 8–12 kDa belonging to the chemotactic cytokine family) play a pivotal role in shaping the TME by directing the recruitment and spatial distribution of immune cells (Ozga et al., 2021). The chemokine system implicated in tumorigenesis comprises approximately 50 ligands, 20 signalling Gαi-protein-coupled seven-transmembrane receptors (GPCRs), and four atypical chemokine receptors (ACKRs).

Solid tumours are typically composed of two major compartments: the tumour parenchyma, consisting of cancer cells, and the tumour stroma, containing non-malignant components, such as fibroblasts, endothelial cells, and immune cells. Chemokines influence compartments regulating stem-like properties, proliferation, and invasiveness of tumour cells, while promoting neoangiogenesis, neurogenesis, and fibrogenesis within stromal cells. This highlights their multifaceted role in tumour immunity (Ozga et al., 2021).

To understand chemokine-mediated mechanisms, four key aspects should be considered: (i) receptor type, (ii) chemokine ligands, (iii) cell expression, and (iv) general immune function. For instance, ACKR1 interacts with chemokine ligands, such as CXCL1–3, CXCL5–8, CXCL11, CXCL13, CCL2, CCL7, CCL14, CCL17, CCL22 and is expressed mainly on endothelial as well as red blood cells, mediating chemokine scavenging and transcytosis. In contrast, ACKR2 shares some ligands (e.g., CCL2, CCL7, CCL14, CCL17, CCL22) and additional ones (CCL3–5, CCL8, CCL11), but is expressed on lymphatic endothelium, dendritic cells, and B cells, functioning primarily in chemokine scavenging. Similarly, CCR2 regulates type 1 adaptive immunity and monocyte trafficking through its expression on endothelial cells, Th1 cells, NK cells, immature dendritic cells, basophils, monocytes, and macrophages, while CCR3 is more closely linked with type 2 adaptive immunity (Ozga et al., 2021).

Two master regulators, transforming growth factor β (TGF-β) and signal transducer and activator of transcription 3 (STAT3), govern many pathological processes in cancer (Bayik and Lathia, 2021). These factors orchestrate communication with cancer stem cells (CSCs). TGF-β stimulates CSC phenotypes, promotes immunosuppressive myeloid cells, polarizes Treg and Th17 cells, and suppresses cytotoxic T and NK cell activity. STAT3, in turn, maintains CSCs, generates Treg cells, suppresses cytotoxic T cells, and drives polarization of tumour-associated macrophages (TAMs).

Notably, microbial dysbiosis and TME is various across different types of tumours (Wei et al., 2025; Liu et al., 2024; Bautista et al., 2025d). Among others, this microbial heterogeneity depends on the localization, for instance upper/lower part of gastrointestinal tracts. The microbiome contributes to tumourigenesis by inducing genomic instability, mutations, and epigenetic modifications including DNA methylation, histone changes, and chromatin remodelling (Cao et al., 2024; Nobels et al., 2025). Microbes can silence tumour suppressor genes or activate oncogenes, thus promoting dysbiosis-associated carcinogenesis. For example, *F*. *nucleatum* plays a well-established role in colorectal cancer (CRC) development through several mechanisms: (1) activation of TLR4 signalling, leading to NF-κB activation, upregulation of microRNA-21, and induction of MyD88-driven autophagy in cancer cells; (2) expression of the virulence factor FadA, which binds E-cadherin, increasing annexin A1 expression and upregulating c-Myc and cyclin D1; (3) immune evasion through the outer membrane protein Fap2, which binds the inhibitory TIGIT receptor on NK and T cells; (4) downregulation of anti-tumour adaptive immunity, correlated with reduced CD3^+^ T-cell infiltration within the TME (Matson et al., 2021; Zhao et al., 2023).

Notably, c-Myc is a central oncogene with wide relevance across cancers (Ye et al., 2024). Jiang X. et al. (2024) recently demonstrated that circular RNA hsa_circ_0000467 enhances CRC progression by promoting eIF4A3-mediated c-Myc translation. Circular RNAs (circRNAs), a class of non-coding RNAs, regulate tumourigenesis by modulating cancer growth and immune pathways; for instance, circRNA_0000392 promotes CRC malignancy. Mechanistically, hsa_circ_0000467 binds eIF4A3 and c-Myc mRNA to form cytoplasmic complexes that facilitate c-Myc translation, subsequently downregulating E-cadherin and promoting CRC invasion and metastasis (Jiang H. et al., 2024).

The TME comprises a diverse array of elements, including endothelial cells, cancer-associated fibroblasts, pericytes, adipocytes, neurons, and immune cells within a dynamic network of microvessels (de Visser and Joyce, 2023; Valkenburg et al., 2018; Elia and Haigis, 2021; Cullin et al., 2021; Aliazis et al., 2025). The intra-tumoural microbial microenvironment (ITMM) additionally includes microorganisms residing in solid tumours, which are typically hypoxic, acidic, and characterized by elevated interstitial fluid pressure (Lin et al., 2021). A notable example is Gammaproteobacteria detected in pancreatic ductal adenocarcinoma, which metabolizes gemcitabine (pyrimidine nucleoside analogue) into an inactive form, thus inducing chemoresistance (Kaźmierczak-Siedlecka et al., 2023a). This underscores the bidirectional interactions between intra-tumoural microbes and anticancer drugs.

Tumour immunity represents a complex network of interactions between immune and tumour cells (Wang J. et al., 2025). Considering chemokines, it should be emphasized that they can provide, either pro- or anti-tumourigenic effect. Chemokines participate in recruiting immune cells into TME leading to the forming them into pro-tumourigenic stage (Xavier et al., 2025; Mempel et al., 2024). Based on the presence or absence of ELR (Glutamic acid–Leucine–Arginine) motif regions, CXC chemokines are classified into two groups: ELR^+^ and ELR^−^ chemokine ligands. It is significant due to their different functional roles, i.e. the first one promotes angiogenesis and the other mediates anti-angiogenesis (ligands CXCL9 - 11, receptor CXCR3, immune cells: NK, Th1, Treg, CD8^+^ T, dendritic cells). Notably, inducible chemokines (inflammatory) expressed only in pathogenic conditions. It is needed to promote the expression of chemokines recruiting anti-tumoural immune cells (Xavier et al., 2025). Immune cells in the TME are broadly divided into adaptive (T and B cells) and innate cells (monocytes/macrophages, NK cells, dendritic cells, neutrophils, eosinophils, and basophils) lineages. These cells originate from hematopoietic stem cells through two progenitor pathways: (1) common myeloid progenitors, giving rise to eosinophils, basophils, neutrophils, monocytes/macrophages, and dendritic cells; (2) common lymphoid progenitors, producing T cells, B cells, and innate lymphoid cells (including NK cells). Immature myeloid cells, myeloid-derived suppressor cells (MDSCs), also contribute to the immunosuppressive TME (Sia et al., 2022). Adaptive immune cells mount antigen-specific responses, whereas innate immune cells mediate non-specific defense mechanisms (Wang Q. et al., 2025). The immune landscape of tumours often exhibits dominance of pro-inflammatory macrophages (M1 type) (Nicolini and Ferrari, 2024). Within the tumour milieu, however, M1 macrophages may undergo polarization into tumour-associated macrophages (TAMs) or immunosuppressive M2 macrophages under the influence of cytokines such as IL-4, IL-6, IL-10, and TGF-β. M2 macrophages promote tumour progression through secretion of immunosuppressive mediators and metabolic reprogramming marked by enhanced oxidative phosphorylation, increased reactive oxygen species, and mitochondrial damage (Nicolini and Ferrari, 2024). Cancer-associated fibroblasts are another essential component of the TME. They promote tumour progression and metastasis, partly through uptake of exosomal miR-146a-5p and miR-155-5p, which enhance CRC metastatic potential (Zhong et al., 2024). Exosomes-related aspects of microbial influence on anti-cancer therapeutics are discussed in the Experts’ Opinions section. Microbes can also modulate the tumour immune microenvironment. For instance, *F. nucleatum* affects CD11b+ myeloid cells, TAMs, and dendritic cell populations (both classical and CD103+ regulatory DCs, thereby promoting tumourigenesis) (Cao et al., 2024). In oral squamous cell carcinoma, *F. nucleatum* enhances tumour progression *via* metabolic regulation upregulating glucose transporter 1 (GLUT1) and accumulating lactic acid through activation of the GalNAc–Autophagy–TBC1D5 signalling axis (Cao et al., 2024).

## 3 Cancer types and microbiota

As it was mentioned above, dysbiotic microbial signature is various considering different types of cancers. Among others, it depends on the localization of tumour and its stage. Additionally, the negative influence of anti-cancer management is also observed, especially in case of chemotherapy/radiotherapy. Head and neck cancers are often treated with radiation causing side effects, such as oral dysbiosis and mucositis (Makarewicz et al., 2024). Below, there are presented changes of microbiota in main cancers.

There are some key microbial drivers in gastric cancer (besides *Helicobacter pylori*), such as *Fusobacterium nucleatum* (found in gastric tumour tissue and mediates immune evasion), *Streptococcus anginosus*, and *Propionibacterium acnes* (in *H. pylori*-negative gastric cancer) (Bautista et al., 2025c). It is known that gastric dysbiosis is induced by *H. pylori* infection, however, its eradication (supported also by probiotics, which are able to positively modify microbiota) can restored microbial imbalance. Above listed, pathobiont *S. anginosus* interacts with host cells, alters TME, and promotes gastric tumourigenesis. Bacteria act by their virulence factors. The activation of pro-tumourigenic mitogen-activated protein kinase is caused by binding of surface protein from *S. anginosus* (TMPC) to the receptor on host cells (annexin A2) (Zeng et al., 2024). Notably, not only bacteria are involved in carcinogenesis, but also fungi and viruses (Kaźmierczak-Siedlecka et al., 2022b). In gastric cancer samples, the increased levels of *Candida albicans* and *Candida tropicalis* have been detected. Other fungal dysbiosis regards the enrichment of *Cutaneotrichosporon*, *Malassezia*, *Archaeorhizomyces*, and *Solicoccozyma*. These dysbiotic changes affect the functioning of immune system, cytokine and chemokine signalling. Epstein-Barr virus is involved in gastric carcinogenesis. It induces the hypermethylation of host cells (Zeng et al., 2024). Gut microbial alterations are different in CRC patients. It is associated with the localization of tumour and another activity of microbiome in intestines. There are several main pathogens related to CRC, such as *Streptococcus bovis*, *F. nucleatum* (its role has been explained above), *Enterococcus faecalis*, *Escherichia coli*, *Peptostreptococcus anaerobius* as well as enterotoxigenic *Bacteroides fragilis* (Cheng Y. et al., 2020a). Moreover, the composition of microbiome is different in early and advanced stage of CRC. Microbes are involved in development of CRC by multiple mechanisms. For instance, *E. faecalis* forms superoxide leading to the DNA damage to intestinal epithelial cells. Enterotoxigenic *B. fragilis* produces toxin and promotes colon tumourigenesis by elevating STAT3 and Th17 response (Kim and Lee, 2020). Considering microbiome related aspects and pancreatic cancer (as a part of digestive system) it should be emphasized that there is a strong connection between this tumour risk and oral microbial alterations. Recently, in 2025, it has been demonstrated that genus *Candida* as well as periodontal pathogens, such as *Porphyromonas gingivalis*, *Eubacterium nodatum*, and *Parvimonas micra* are correlated to increased risk of pancreatic cancer (Meng et al., 2025). Besides, oral dysbiosis, the changes are also observed in gut microbiome regarding loss of microbial diversity (Li et al., 2021).

Gut microbiome is implicated in the pathogenesis of melanoma (Makaranka et al., 2022). The abundance of Saccharomytecales and *Prevotella copri* species is observed in melanoma patients (Vitali et al., 2022). Microbiome is also involved in the response to immunotherapy based on immune-checkpoint inhibitors (ICIs) (Fortman et al., 2023). Notably, a significant higher abundance of Ruminococcaceae in responders is found compared to nonresponders treated with anti-PD-1 (or other systemic therapy) (Spencer et al., 2021). The modulation of gut microbiome for better outcomes with immunotherapy can be obtained by high-fiber diet, which results in increased overall survival and decreased therapy related side effects (Haddad and Holder, 2024). Microbiome can also promote the development of prostate cancer (Sfanos et al., 2018). There is the functional axis created by microbial microbes found in gut, urinary tract, and prostate. It was demonstrated that the increased level of some microorganisms, such as *Bacteroides*, *Ruminococcus*, and *Akkermansia* results in the promotion of epithelial proliferation as well as immune evasion. Moreover, the reduce amount of anti-inflammatory taxa (*Faecalibacterium*, *Bifidobacterium*) reduces the function of Treg cells and impairs mucosal barrier integrity contributing to tumour progression. To obtain better clinical outcome, some microbiota modulators can be introduced. For instance, *Akkermansia muciniphila* provides beneficial effects regarding the enhancement of anti-tumour immunity. It improves the response to androgen deprivation therapy (Bautista et al., 2025a).

## 4 Bacterial-mediated cancer immunotherapies and non-immune checkpoint blockade immunotherapies

Given that tumour immunity is strongly modulated by microbiome, certain bacterial species have emerged as promising targets or adjuvants in cancer immunotherapy. Microorganisms can exert both stimulatory and suppressive effects through virulence factors, structural components, and diverse metabolites that shape immune cell activity. Two major ICIs currently used in in oncology target the PD-1/PD-L1 and CTLA-4/CD80/86 pathways. These checkpoints function as negative co-stimulatory receptors, regulating T-cell activation and maintaining immune tolerance (Sia et al., 2022). The U.S. Food and Drug Administration (FDA) has approved the following ICIs, such as ipilimumab, tremelimumab, nivolumab, pembrolizumab, cemiplimab, atezolizumab, avelumab, durvalumab (Arafat Hossain, 2024).

Clinical response to ICIs is modulated by the gut microbiota (Fernandes et al., 2022; Li et al., 2019; Peng et al., 2020). For instance, *Bifidobacterium pseudolongum* enhances anti–PD-1 and anti–CTLA-4 immunotherapy in mouse model of cancer by producing inosine, which binds to adenosine 2A receptors (A2ARs) on CD4^+^ T cells, promoting their activation and augmenting anti-tumour immunity (Yang et al., 2024; Mager et al., 2020). Similarly, *Bacteroides fragilis* enhances the efficacy of anti–CTLA-4 therapy by inducing Th1-type immune responses in lymph nodes and promoting the maturation of intratumourous dendritic cells (DCs) (Cheng W. Y. et al., 2020). Bacteria-mediated cancer immunotherapies (BMCIs) can induce tumour regression through mechanisms, such as metabolic modulation, apoptosis induction, and immune activation (Kwon et al., 2024). These strategies are increasingly recognized as valuable components of precision oncology. Two key considerations for future development include: (1) bacterial specificity–distinct microbial species or strains may synergize differently with specific immune-checkpoint inhibitors, (2) mechanistic understanding–elucidating the molecular pathways by which microbes influence immune responses is essential to optimize therapeutic outcomes. Beyond checkpoint inhibition, the microbiome also affects non-checkpoint immunotherapies, including immune-cell vaccines and adoptive cell therapies, such as dendritic cell (DC) vaccines, tumour-infiltrating lymphocytes (TILs), and adoptive T-cell transfer (Newsome et al., 2022; Lee et al., 2021; Adamik et al., 2023). DCs are professional antigen-presenting cells responsible for processing and presenting tumour-derived peptides and proteins to T and B lymphocytes. The goal of DC-based vaccination is to stimulate tumour-specific cytotoxic T cells capable of eliminating cancer cells, while maintaining low systemic toxicity (Mody et al., 2015). The generation of DC vaccines typically involves three main steps (Gardner et al., 2020): (1) isolation of monocytes or immature DCs from peripheral blood; (2) differentiation and activation–monocytes are converted into immature DCs using IL-4 and GM-CSF, or immature DCs are matured with specific cytokine cocktails and tumour-associated antigens (e.g., peptides or tumour lysates); (3) re-infusion of tumour-specific DCs into the patient to elicit a targeted immune response. Personalized DC-based vaccines targeting tumour neoantigens (unique non-self peptides arising from somatic mutations) represent a promising next-generation immunotherapy with high tumour specificity and immunogenicity (Kamigaki et al., 2024; Ding et al., 2021). Interestingly, Li X. et al. (2024) demonstrated a method allowing to prepare neoantigen-reactive T cells to enhance the efficacy of adoptive cell transfer in mice model study. It has been prepared regarding immunization with a tumour lysate-loaded DC vaccines. Moreover, the results of phase I trial indicated that neoantigen-targeted DC vaccination is safe in case of non-small cell lung cancer (Ingels et al., 2024). Although non-checkpoint immunotherapies hold great promise, their use as monotherapy remains limited, and combination approaches are likely to offer greater benefit. Moreover, it appears that microbiome composition and function significantly influence their efficacy, warranting detailed mechanistic and translational studies.

## 5 Interaction between microbiota-derived metabolites and the immune system

Microorganisms continuing the microbiome participate in diverse processes central to tumour biology including epigenetic regulation, genomic stability, metabolic reprogramming, tumour immunity, inflammatory signalling, and metastasis. Their effects are mediated through both virulence factors and microbiota-derived metabolites, which can be anti- or pro-tumourigenic depending on context (Elinav et al., 2019; Cong et al., 2024). Several mechanisms illustrate how these metabolites influence tumour–immune interactions: (1) Enterotoxigenic *Bacteroides fragilis* promotes colonic inflammation by inducing DNA damage and IL-17A production *via* Th17-cell activation; (2) Gallic acid, a microbiome-derived metabolite, can inhibit the tumour-suppressive activity of mutant TP53, thereby promoting tumour progression; (3) *Flavonifractor plautii* degrades beneficial anti-cancer flavonoids, diminishing their chemoprotective potential (Mima et al., 2023). These examples highlight the dual nature of microbiota-derived metabolites, which can either sustain anti-tumour immunity or promote immune evasion. Understanding this metabolic–immunologic interface is essential for the development of metabolite-based adjunct therapies in oncology. To explain this aspect more precisely, a concise summary of key metabolites (SCFAs, bile acids, tryptophan) and their bidirectional interactions with immunological pathways in cancers (pancreatic tumours, CRC) is presented in Table 1.

**TABLE 1:** The most important metabolites with their bidirectional interference with immunological pathways in cancers (pancreatic tumours, CRC). There are presented different types of studies in which the influence of particular microbiome-related metabolites affects the functioning of immune system and *vice versa*.

References	Microbial community	Metabolites	Type of cancer	Type of study	Design	Conclusions
Nomura et al. (2020)	Intestinal microbiome	Short chain fatty acids (SCFAs)	Solid tumours, including nine patients with tumours directly related to the digestive system	Prospective cohort study	The study included 52 patients treated with nivolumab or pembrolizumab. The patients were divided into two groups. The division was made based on response to treatment, in accordance with the Response Evaluation Criteria in solid tumours. SCFAs concentrations were examined in stool and plasma samples prior to PD-1 inhibitor administration and measured by ultraperformance liquid chromatography.	The presence of SCFAs in stools is associated with PD-1 treatment and longer survival; These effects are due to the presence of acetate, propionate, butyrate and isovalerate, which are detected in high concentrations in faeces and plasma, respectively. SCFAs have immunomodulatory functions, influencing CD4+ lymphocytes and antigen-presenting cells; Among SCFAs, butyrate is particularly responsible for reducing inflammation by promoting tolerance in CD4+.
Ternes et al. (2022)	*Fusobacterium nucleatum*	Formate	Colorectal cancer (CRC) (HT-29 cells were administered subcutaneously to the groin area of mice)	Experimental study	Cultured CRC cells and stool samples were collected from 115 patients and subjected to preliminary analysis. Next, CRC tumours were implanted into mouse models and formate was administered. Some of the mice had previously received formate and some were colonised with *F. nucleatum*.	Formate indirectly stimulates the invasiveness of CRC cells via AhR; Formate promotes the expression of Th17 cells, which may play an important role in cancer development; *F. nucleatum* affects the expression of CD4+ and IL-17+ T cells.
Hezaveh et al. (2022)	Four strains of *Lactobacillus* (*L. reuteri*, *L. intestinalis*, *L. johnsonii*, *L. murinu*)	Tryptophan derivatives (Indole-3- acetic acid (IAA), Indole-3- lactic acid (ILA)	Pancreatic cancer in a mouse model (mT4 mouse pancreatic cancer (KPC) lines were implanted directly into the mouse model)	Preclinical experimental study	Four *Lactobacillus* strains were isolated from the mouse intestine and identified using 16S PCR. The bacteria were then cultured in the presence and absence of tryptophan and the resulting metabolites analysed using metabolomics. Next, the bacteria were administered to appropriate mice that had been implanted with pancreatic cancer cells. Immune markers and AhR receptor activation were then compared	*L. reuteri* and *L. murinis* are particularly responsible for producing IAA and ILA, which activate AhR in tumour macrophages and increase the risk of cancer growth; *L. murinis* is a special strain: in its presence, there is increased expression of Arg1, IL-10; The absence of IAA and ILA indirectly increases IL-1β expression and activates CD8+ T cells
Cong et al. (2024)	Intestinal microbiome	Bile acids (especially deoxycholic bile acid DCA, licholic acid LCA)	CRC MC38 line cells were injected into mice	*In vivo* experimental study	Bile acid metabolites were identified, with a particular focus on DCA. The influence of altered bile acids on tumour growth and their effect on the immune system were then studied in a mouse tumour model.	DCA inhibits the function of CD8+ T cells in colorectal cancer; LCA inhibits TNF-α and INF-γ expression.
Mowat et al. (2023)	Intestinal microbiome	SCFAs mainly butyrate and propionate	CRC MC38 with additional OVA modification was administered into the wall of the descending colon	Experimental study	CRC samples were collected from patients, processed, and cultured. The CRC was cultured in mouse models and treated with butyrate, propionate, or a combination of the two for 24–48 h. The patient and mouse samples were analysed.	Butyrate and propionate increase the ability of CRC to activate CD8+ T cells; Butyrate and propionate SCFAs enhance T-cell activation by indirectly stimulating feedback between CRC and CD8+ cells; Butyrate and propionate SCFAs increase MHCI expression.

## 6 Immunonutrition

The treatment of cancer requires a multidisciplinary approach, and microbiome-oriented strategies. Nutrition management should follow several key steps: screening and assessment, diagnosis, intervention, monitoring, and evaluation (Martinelli et al., 2023). Within this framework, immunonutrition (the targeted modulation of the immune system through specific nutrients) represents a valuable adjunct to immunotherapy, particularly in the context of gut and tumour microbiota interactions (Yang et al., 2024; Lee et al., 2020; Dübüş et al., 2023; Weimann, 2018; Nami et al., 2023; Tang et al., 2022; Elkrief et al., 2025; Nakatsu et al., 2024). Interestingly, the nutritional status can be used as prognostic tool (among others, to assess overall survival) in patients receiving immunonutrition (Mattavelli et al., 2025). For instance, low Prognostic Nutritional Index (PNI) is associated with the prediction of decreased both overall survival and progression-free survival in patients with advanced head and neck squamous cell carcinoma (Guller et al., 2021). However, despite promising preclinical findings, clinical data on microbiome-modulating immunonutrients remain limited. This section outlines the bidirectional relationships between the microbiome (including tumour-resident microbiota) and immunonutrition.

### 6.1 Core principles of immunonutrition

Optimal nutritional intervention is especially critical in the perioperative period for cancer patients to preserve the integrity of the mucosa-associated microbiome, prevent increased intestinal permeability, and limit inflammation. Four major immunomodulatory nutrients are routinely used in cancer care: arginine - enhances lymphocyte proliferation and wound healing; glutamine - supports enterocyte function and immune responses; omega-3 fatty acids - exhibit anti-inflammatory and anti-cachectic effects; nucleotides - promote immune cell proliferation and repair mechanisms. The term immunonutrition thus denotes nutritional formulas enriched with bioactives substrates capable of modulating both innate and adaptive immunity. Interactions among immunonutritions, the immune system, intestinal immunity, and the microbiome are increasingly recognized as central to systemic immune competence and tumour biology.

### 6.2 Clinical evidence linking immunonutrition and the gut microbiome

Recently, we have evaluated (Kaźmierczak-Siedlecka et al., 2025) the effects of preoperative immunonutrition on the gut microbiome of patients with colorectal and gastric cancer. Participants were randomized to receive either an immunonutrient-enriched formula or a standard diet for 7 days prior to surgery. Stool samples were preserved in DNA/RNA Shield Fecal tubes, and microbial composition was analysed using 16S metagenomic sequencing (V3-V4 regions; Illumina MiSeq, 300 bp paired-end reads). Although overall microbial community compositions did not differ significantly between groups, nominally significant shifts were observed (p < 0.05) in taxa such as *Bilophila*, *CAG-56*, *Clostridium sensu stricto 1*, *Coprobacter*, *Holdemania*, *Fusicatenibacter*, *Ruminococcus*, and *[Eubacterium]* (Kaźmierczak-Siedlecka et al., 2025). Notably, these results should be considered carefully due to limited statistical power. It can be associated with several factors, such as small sample size, short time of intervention, uncertainty about compliance with consuming study products. Lee et al. (2023) reported results from a secondary analysis of a randomized controlled trial investigating preoperative immunonutrition in colon cancer. Patients received either immunonutrition plus standard diet or standard diet alone for 7 days before surgery (using Newcare Omega®, Daesang Life Science, Korea; 400 mL/day; per 100 mL: 100 kcal, 5 g protein, 3 g fat, 13.75 g carbohydrate, 0.25 g arginine, 0.23 g omega-3 fatty acids). At the phylum level, no significant differences were observed (Firmicutes: 69.1% vs. 67.5%, p = 0.624; Bacteroidetes: 19.3% vs. 18.1%, p = 0.663; Actinobacteria: 6.7% vs. 10.6%, p = 0.080). However, at the genus level, *Faecalibacterium* and *Prevotella* were more abundant, while *Clostridium* spp. were less abundant in the immunonutrition group—though differences did not reach statistical significance (D’Ignazio et al., 2020). Despite the fact that there is no clear evidence confirming that immunonutrition affects the overall composition of gut microbiome *per se*, nevertheless, initial alterations (even without statistical power) can suggest its potential impact on microbial community regarding also the activity of intestinal microbes. Interestingly, immunonutrition may modulate not only gut microbiome but also the tumour microbiome. D’Ignazio et al. demonstrated that preoperative oral immunonutrition altered the tumour immune microenvironment in gastrointestinal cancer patients (n = 16 immunonutrition; n = 8 standard diet) (D’Ignazio et al., 2020). Immunohistochemical analysis of tumour specimens revealed increased infiltration of CD4^+^ helper and CD8^+^ cytotoxic T lymphocytes, along with modulation of markers, such as PD-1, PD-L1, FOX-P3, CD68, and CD163. These findings suggest that immunonutrition can enhance both cellular and humoral immune responses, reshaping the tumour immune landscape (D’Ignazio et al., 2020).

Collectively, current evidence supports the role of immunonutrition in reducing perioperative infectious complications, improving nutritional and inflammatory status, enhancing immune function, promoting apoptosis, and mitigating chemotherapy-related side effects (Martinelli et al., 2023). Nevertheless, large-scale, multi-omics-driven clinical trials are needed to elucidate the precise microbiome-mediated mechanisms underlying these effects.

## 7 Expert perspectives and future directions

Statement 1 The bidirectional link between the immune system and microbiome – challenges for drug design.

A growing body of evidence indicates a tight correlation between microbial metabolic pathways and the efficacy of anti-tumour therapies (Li Z. et al., 2024; Polak et al., 2024; Kao et al., 2022; Schmitt and Greten, 2021). This relationship opens new opportunities for developing drugs that target specific tumour–microbiome metabolic axes. The tumour immune microenvironment influences the recognition and elimination of malignant cells through cytokine, interleukin, and antibody-mediated pathways, all of which may be affected by microbial metabolites. Understanding these immune–microbial interactions is essential for designing microbe-based anti-cancer agents that selectively target molecules or pathways involved in tumour progression and immune modulation. Several microorganisms are known to elicit antigen-specific T-cell responses, making antigen-engineered commensal bacteria a particularly promising strategy. For example, the skin commensal *Staphylococcus epidermidis* has been genetically modified to express antigens derived from murine melanoma cells, successfully triggering robust T-cell-mediated responses against both subcutaneous tumours and lung metastases (Sajjath et al., 2023). Notably, *S. epidermidis* is able to exert direct anti-tumour effect (Bautista et al., 2025d). Another emerging concept involves the use of bacteriophages to selectively eliminate pathogenic bacteria within tumours, thereby acting as intratumoural microbial modulators. Nevertheless, key parameters (such as phage type, dosing, and delivery route) must be precisely optimized to ensure efficacy and safety (Cao et al., 2024). Overall, designer drugs based on microbiome components, genetically engineered microbes, or synthetic microbial analogues hold strong translational potential. However, their clinical application will require extensive pre-clinical validation and phased clinical studies to confirm safety, immunogenicity, and long-term outcomes. Multiple variables and factors should be involved in the process of designing drugs based on bidirectional interaction between immune system and microbiome, such as PD-L1 expression, MSI-H (microsatellite instability high), tumour mutational burden (both of them related to increased generation of tumour-associated antigens), neutrophil-to-lymphocyte ratio. These factors are recognized as immune-relevant biomarkers in oncology (prognostic and predictive) (Kroemer et al., 2024). It should be emphasized that manipulating both microbiome and TME is one of the strategy to improve the effect of anti-cancer management, however complexity and heterogeneity of them cause that it is still challenging in cancer approaches (Nie et al., 2025; Bautista et al., 2025b). The individual profile of microbiome should be taken into consideration, especially in the context of personalized medicine.

Statement 2 Tumour organoid-immune co-culture models as a tool to improve immunotherapy.

Tumour organoids, generated from patient-derived or tumour-cell cultures, can reproduce the morphological, genetic, and functional heterogenity of primary tumour (Wang J. et al., 2025; Polak et al., 2024; Gavrielatou et al., 2020). These models provide unique opportunities to study tumour initiation, drug response prediction, and personalized drug screening. Importantly, when organoids are co-cultured with immune cells, they allow direct visualization of tumour–immune interactions, revealing how immune cells recognize and eliminate malignant cells and how tumours evade immune surveillance. Integrating 3D bioprinting with organoid-immune co-culture systems further enhances the physiological relevance of these models. 3D bioprinting enables the precise spatial reconstruction of tumour architecture including stromal and vascular components, thus creating a more accurate *in-vitro* representation of the TME. This combined approach can facilitate detailed analyses of immune infiltration, signalling dynamics, and therapeutic response, accelerating the translation of experimental findings into clinical practice. Patient-derived organoids: immune cells co-culture models can be applied to precision medicine regarding personalized expansion of lymphocytes for adoptive cell transfer therapies (cytotoxic NK cells, tumour reactive T cells), screening of immune checkpoint therapies as well as screening of bi-specific antibodies (Magré et al., 2023). Microbiome could be integrated in above mentioned aspects, however, data are strongly limited.

Despite these advantages, significant standardization challenges remain. Issues, such as culture stability, reproducibility, accurate drug-sensitivity testing, and consistent immune-system activation limit widespread implementation. Notably, low efficiency of organoid generation from tumour tissue (with average level of organoid generation as 36.8%) is one of the limitation. Moreover, it has been observed that in high-throughput drug screening, low numbers of organoid establishment complicate their use (Magré et al., 2023). Addressing these challenges through protocol harmonization and cross-platform validation is essential for realizing the full potential of organoid-based immuno-oncology research (Wang Q. et al., 2025).

Statement 3 Predicting patients’ resistance to anti-cancer drugs as part of precision medicine.

Resistance to ICIs remains a major clinical barrier. It is frequently driven by alterations in the TME, low checkpoint expression, and immune-evasion mechanisms (Arafat Hossain, 2024). Although several predictive biomarkers have been proposed (particularly for PD-1/PD-L1 blockade monotherapy) their accuracy remains limited in combination regimens. For instance, in combination therapy with atezolizumab (anti-PD-L1) and bevacizumab (anti-VEGF), high T-effector gene-signature expression and myeloid inflammation are associated with prolonged progression-free survival (Yang et al., 2024). Another combination strategy, such as anti-PD-1 + fecal microbiota transplantation (as microbiome modulation) restores ICIs sensitivity as well as improves patients’ stratification (Kyriakidis et al., 2025). Epigenetic regulation also contributes to therapeutic resistance: overexpression of miR-200 suppresses PD-L1 expression, thereby impairing PD-1-targeted therapy. Moreover, an interferon-γ gene signature has been identified as both a prognostic and predictive marker of response to immunotherapy (Gavrielatou et al., 2020). An emerging field pharmacomicrobiomics adds further complexity. Inter-individual variability in both microbiome composition and tumour microenvironment can profoundly affect drug metabolism and response (Kaźmierczak-Siedlecka et al., 2023b; Ryu et al., 2021; Blake et al., 2024; Zhou et al., 2021). Integrating microbiome profiling into precision-medicine pipelines could thus help anticipate resistance and tailor immunotherapeutic strategies to individual patients. Notably, precision-medicine pipelines in the context of microbiome should regard not only microbiome *per se*, however also other -omics. It is also recommended to analyse both targeted and untargeted metabolomics. Additionally, different methods of microbiome analysis should be used (shallow shotgun, 16S) to provide deep insight into microbiome profile regarding various levels of microorganisms classifications. Microbial-based biomarkers can be useful in the prediction of patients’ resistance to anti-tumour medications. In case of cyclophosphamide, anti-cancer immune response can be shaped by gut microbiome (Viaud et al., 2013).

Statement 4 Engineered exosomes, engineered bacteria, and therapeutic strategies.

Engineered exosomes represents a novel class of artificial extracellular vesicles designed to deliver nucleic acids, peptides, and small-molecule drugs to target cells (Qiu et al., 2024). Exosomes are secreted by nearly all cell types (including tumour cells) and play central roles in intracellular communication. One notable microbial target in exosome research is *F. nucleatum*, which promotes which promotes colorectal-cancer (CRC) progression and metastasis through non-coding RNA modulation. *F. nucleatum* infection upregulates microRNA-21 and long non-coding RNA EVADR, and enhances exosomal secretion of miR-122-5p, a tumour-suppressor microRNA (Zhang et al., 2023). Loss of intracellular miR-122-5p, mediated by hnRNPA2B1-dependent export, activates the FUT8/TGF-β1/Smads signalling axis, thereby facilitating CRC cell migration and invasion. Consequently, targeting microbial infections or their downstream exosomal pathways could represent a novel anti-metastatic strategy.

**FIGURE 1:**
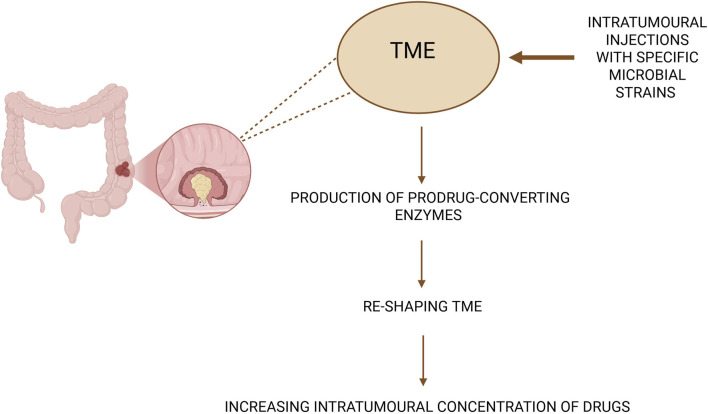
Concept of TME re-shaping through intratumoural bacterial injections. These types of injections with specific microbial strains (for instance, above mentioned *S. typhimurium*) lead to the production of prodrug-converting enzymes causing re-shaping TME, which allows to obtain higher concentration of chemotherapeutics in intratumoural environment. This conception is strongly associated with the programming microorganisms to act as an activator of medications within tumours. Own elaboration based on literature (Cao et al., 2024). This figure has been created using Biorender.com.

Engineered bacteria also offer promising therapeutic applications. Microbes can be programmed to produce pro-drug-converting enzymes, enabling local activation of chemotherapeutics within tumours. For example, intratumoural injection of attenuated *Salmonella typhimurium* (VNP20009) expressing *Escherichia coli* cytidine deaminase markedly increased intratumoural concentrations of active 5-fluorouracil (Cao et al., 2024). The potential actions of engineered bacteria encompass four domains: pro-drug enzyme production (e.g., cytidine deaminase systems), targeting tumour stroma, reprogramming macrophage polarization (increasing M1-like, decreasing M2-like populations), and controlled expression of cytotoxic mediators. Such approaches provide a conceptual basis for TME re-shaping *via* bacterial injections, illustrated in Figure 1.

Statement 5 SRT in microbiologically driven cancer research.

SRT comprises methods that map gene-expression profiles within intact tissue architecture, offering unprecedented insight into the spatial heterogeneity of tumour and immune cells (Wang et al., 2023; Cheng et al., 2023; Zheng and Fang, 2022). Two principal SRT approaches are currently used: (1) imaging-based technologies, such as seqFISH+, providing subcellular resolution through cyclic imaging of pre-defined targets (fresh-frozen tissues); (2) next-generation sequencing-based technologies, such as 10x Visium, offering multicellular-level resolution with an untargeted transcriptome readout applicable to fresh-frozen or FFPE tissues. While 10x Visium captures transcripts from multiple cells per spot (limiting single-cell resolution) it still provides valuable spatial transcriptome context unavailable from conventional bulk or single-cell RNA sequencing (Wang et al., 2023; El Tekle et al., 2024). SRT enables comprehensive analysis of tumour microenvironment composition, spatial heterogeneity, and intercellular communication networks (Maniatis et al., 2021; Zhu et al., 2024).

**FIGURE 2:**
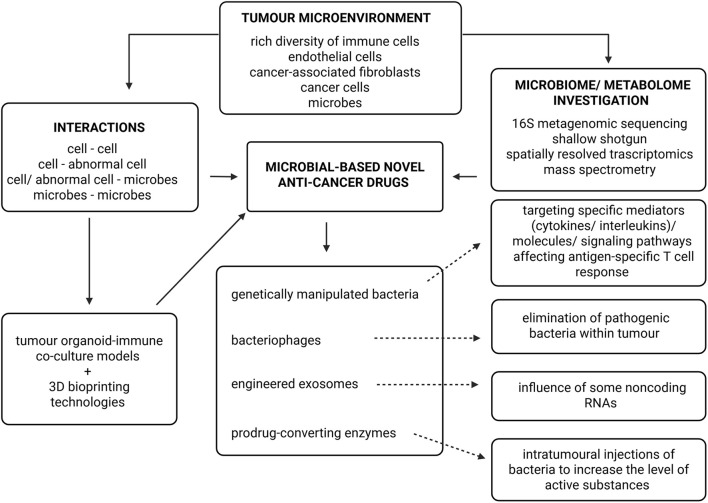
Conceptual framework for microbial-based anti-cancer drug design informed by intratumoural composition. It regards four main areas: genetically manipulated bacteria, bacteriophages, engineered exosomes, prodrug-converting enzymes. The designing microbial-based anti-cancer medications is a complicated process in which multiple elements should be taken into consideration, such as interactions between different types of cells and methods of microbiome and metabolome investigations (shallow shotgun, 16S, mass spectrometry, SRT). Mapping of TME-drugs networks can be included to personalized medicine. This figure has been created using Biorender.com.

The conceptual workflow for designing next-generation microbial-based anti-cancer therapeutics, integrating spatial transcriptomics, microbiome mapping, and metabolic profiling is summarized in Figure 2.

## 8 Technical limitations in microbiome studies

There are some methodological limitations occurring in -omics studies affecting the results, such as difficulties regarding taking oral/rectal swabs, using tubes with or without genetic materials stabilizer, low biomass, variability across sequencing platforms, different methods of analysis (shallow shotgun, 16S).

## 9 Conclusion

Microorganisms coexist with a diverse array of immune and stromal cells within the intratumoural microenvironment. A deeper understanding of their molecular interactions, signalling pathways, and histological heterogeneity is crucial for the rational design of next-generation anti-cancer therapies. The integration of intratumoural microbiome with spatial transcriptomics and pharmacomicrobiomics allows to obtain precision medicine. Specific microbial biomarkers should be detected to not only found tumours in early stage but also to predict the response to treatment and potential patients’ resistance to anti-cancer medications. Currently, the data are strongly undiscovered, especially considering combination therapy, i.e. ICI + ICI presenting different mechanisms of action as well as ICI + modifier of microbiome (FMT, next-generation probiotics). Notably, the analysis of TME can contribute to implement targeted strategy against cancer. Identifying microbial species that shape tumour phenotypes remains a major challenge, yet accumulating preclinical data suggest that microbiome-based interventions hold substantial therapeutic promise. Although most studies remain in early developmental phases, microbial-based tumour therapy is emerging as a transformative strategy in modern oncology.
